# Comprehensive atlas of mitochondrial distribution and dynamics during oocyte maturation in mouse models

**DOI:** 10.1186/s40364-024-00672-z

**Published:** 2024-10-16

**Authors:** Xia Hao, Jian Zhao, Kenny A. Rodriguez-Wallberg

**Affiliations:** 1 Department of Oncology-Pathology, Laboratory of Translational Fertility Preservation, Karolinska Institutet, Stockholm, Sweden; 2 Department of Reproductive Medicine, Division of Gynecology and Reproduction, Karolinska University Hospital, Stockholm, Sweden

**Keywords:** Mitochondria, Oocyte, Mitochondrial distribution, Oocyte maturation

## Abstract

**Background:**

Oocytes, the largest cells in mammals, harbor numerous mitochondria within their cytoplasm. These highly dynamic organelles are crucial for providing energy resources and serving as central regulators during oogenesis. Mitochondrial dynamics ensure proper energy distribution for various cellular processes involved in oocyte maturation. Previous studies have used alterations in mitochondrial distribution as a biomarker to assess the oocyte health. However, there are discrepancies between studies regarding mitochondrial distribution profiles in healthy oocytes. Consequently, a comprehensive mitochondrial distribution profile in oocytes during maturation has not been fully characterized. Additionally, there is a lack of objective, quantitative methods to evaluate alterations in mitochondrial distribution profiles in oocytes.

**Methods:**

This study aims to provide an in-depth overview of mitochondrial distribution profiles in mouse oocytes at different maturation stages: germinal vesicle (GV) stage, metaphase I (MI), and mature metaphase II (MII). Freshly collected mouse GV, MI and MII oocytes were stained with MitoTracker Red. Confocal microscopy was used to obtain images of mitochondrial distribution profiles in these oocytes. Using the Imaris software, we reconstructed three-dimensional (3D) surface renderings of each oocyte and quantitatively illustrated the mitochondrial distribution profiles.

**Results:**

At the GV stage, mitochondria in oocytes were evenly distributed throughout the ooplasm. As oocytes progressed to MI and MII stages, mitochondria aggregated and formed clusters, the mean size of mitochondrial clusters and the proportions of clustered mitochondria increased along with the maturation of oocytes.

**Conclusions:**

Our findings reveal that mitochondria in mouse oocytes are highly dynamic, undergoing significant reorganizations during oocyte maturation. We for the first time provided comprehensive mitochondrial distribution profiles in mouse oocytes at the GV, MI and MII stages. These mitochondrial distribution profiles were further quantitatively evaluated. Our methods provide an objective and standardized approach for evaluating alterations in mitochondrial dynamics, which can be used as biomarkers to monitor oocyte conditions during maturation.

## Background

Oocytes, known to be the largest cells in mammals, harbor a substantial number of mitochondria within their cytoplasm [1]. They are essential for energy production through oxidative phosphorylation (OXPHOS) [2–4]. Serving as the energy factories of cells, mitochondria are quite abundant in oocytes. They play a crucial role in various stages of reproduction, including oocyte maturation, fertilization, and embryogenesis [5]. Beyond energy production, mitochondria are pivotal in a host of cellular processes, such as the regulation of Ca^2+^ homeostasis, reactive oxygen species (ROS) signaling, glycolysis, amino acid and fatty acid metabolism, and the control of apoptosis and mitophagy. Consequently, mitochondria profoundly impact all aspects of mammalian reproduction [1, 5].

Somatic cells typically contain hundreds of mitochondria, forming a dynamic network that regulates various cellular processes through movement, fusion, and fission in response to the cellular energy demands and physiological changes. The flexible movement, fusion and fission of mitochondria is collectively known as mitochondrial dynamics, which are regulated by a suite of mitochondria-shape proteins, including the fission proteins DRP1 (Dynamin-Related Protein 1), FIS1 (Mitochondrial Fission 1 Protein), MFF (Mitochondrial Fission Factor), and MIEF1/2 (Mitochondrial Elongation Factor 1/2), as well as the fusion proteins OPA1 (Optic Atrophy Protein 1), MFN1/2 (Mitofusin 1/2) in human [3]. However, unlike in somatic cells, where mitochondria often form interconnected tubules with abundant cristae and exhibit high metabolic activity (which can vary depending on the cell type and its metabolic demands), the mitochondria in mammalian oocytes are inherently round or oval with sparse cristae and lower metabolic activity [4, 6–8]. To compensate for the low individual mitochondrial activity, oocytes significantly increase the number of mitochondria, ensuring sufficient ATP production to meet their high energy demands while keeping the by-product ROS at a low level [9]. Additionally, oocytes rely on mitochondrial fission and fusion to maintain mitochondrial mass and function, ensuring that mitochondria can meet energy demands even under lower metabolic activity [10]. Notably, elevated levels of the fusion proteins MFN1, MFN2 or OPA1 do not induce tubular mitochondria but lead to mitochondrial aggregation (clustering), particularly in the perinuclear region. In contrast, increased levels of the fission protein Drp1 do not significantly affect mitochondrial distribution [6].

During oogenesis, a notable change is that the volume of the oocyte increases approximately 300-fold. The expansion is accompanied by the accumulation of a substantial number of mitochondria. Mitochondria play crucial roles in oocyte maturation, fertilization and early embryonic development [11, 12]. Interestingly, mature oocytes halt producing new mitochondria until the embryo reaches the blastocyst stage [13]. Consequently, any alterations in mitochondrial function, quantity, spatiotemporal distribution, and ultrastructure within the oocyte may potentially impact the developmental competence of mammalian pre-implantation embryos [14]. Studies have shown a strong correlation between reduced mitochondrial DNA (mtDNA) content and fertilization failure as well as this content is consistently reduced with increased ovarian aging [12, 15, 16]. The mitochondrial content in the oocytes of older women or those with diminished ovarian reserve (DOR) is significantly lower compared to their younger counterparts or women with a normal ovarian reserve [17]. However, an adequate number of mitochondria or mtDNA copies is only one aspect of the complex mitochondrial network that ensures oocyte quality. Other factors, such as mitochondrial distribution and dynamics, also play indispensable roles [4]. Therefore, preserving mitochondrial function, structure and spatiotemporal distribution are of paramount importance for oocytes in assisted reproductive technology (ART).

However, mitochondrial research in the scope of reproduction and infertility treatments using ART remains a developing field. Due to the complexity of the mitochondrial network, accurately quantifying the number of mitochondria in cells is often a challenge. Several techniques are currently used for this purpose. Electron microscopy has been used to morphometrically determine mitochondrial number [18, 19], but this method is tedious and expensive. Despite its high resolution, electron microscopy has a limited field of view, making it difficult to get a comprehensive overview of all mitochondria within large cells, such as mammalian oocytes. Consequently, the oocyte mitochondrial mass is frequently indirectly estimated by measuring mtDNA copy number, typically using quantitative real-time PCR [9, 20]. However, each mitochondrion often contains multiple copies of mtDNA, and mtDNA copy number varies between different oocytes and changes during oocyte maturation. This variability partially explained the observed variations of mtDNA copy numbers in oocytes between different individuals, compromising the accuracy of this estimation in representing the actual number of mitochondria in an oocyte. Additionally, flow cytometry can be used for rapid and high-throughput analysis of mitochondrial content in large cell populations [21]. However, this technique has limitations when only few cells, such as oocytes, are available for analysis. Recently, confocal microscopy combined with software has emerged as a powerful tool to analyze mitochondrial number and mass in cells. Mitochondrial number and mass can be assessed by counting the individual fluorescent spots representing mitochondria and measuring the fluorescence intensity of mitochondria [22, 23]. In addition to the quantification of mitochondria or mtDNA copy number, many studies have reported the distribution patterns of mitochondria in both human oocytes [24–27] and mouse oocytes [6, 28–35], these observations documented a wide range of ‘normal’ mitochondrial distribution patterns in their respective normal control groups, resulting in inconsistent profiles. Additionally, the evaluations were often rough and subjective. Differences in mitochondrial aggregation/clustering patterns during oocyte maturation have been reported across different species [27, 28, 36–38].

To gain deeper insights into how mitochondria influence the maturation process of oocytes and their final quality, it is essential to conduct a comprehensive analysis of mitochondrial distribution in normal oocytes during maturation. At present, studies are inconsistent regarding the mitochondrial distribution changes during oocyte maturation. We therefore designed a study combining confocal microscopy and computational constructed three-dimensional (3D) surface renderings to assess the distribution of mitochondria in mouse oocytes and their changes during maturation. Our goal was to establish a comprehensive atlas depicting the normal distribution of mitochondria in mouse oocytes, serving as a reliable biomarker for monitoring the changes in mitochondrial distribution during maturation and for assessing the quality of oocytes.

## Methods

Chemicals and materials used in this study were purchased from Sigma–Aldrich^®^, Thermo Fisher Scientific^®^ or Gibco, otherwise stated at their first appearances.

### Superovulation induction and tissue retrieval

Eight-week-old inhouse bred B6CBA/F1 female mice (*n* = 3) were received intraperitoneal injection of pregnant mare serum gonadotrophin (5 IU, Folligon^®^, MSD animal health, Brussel, Belgium). 50 h later, the mice were received intraperitoneal injection of human chorionic gonadotrophin (5 IU, Chorulon^®^, MSD animal health, Boxmeer, Holland). 13.5 h later, the mice were sacrificed by cervical dislocation. Both ovaries, oviducts and a part of the uterine horn were collected into Leibovitz 15 medium enriched with 10% fetal bovine serum (FBS), 100 IU/mL of penicillin and 100 µg/mL of streptomycin.

All procedures involving mice were conducted at the Preclinical Laboratory of Karolinska University Hospital, Huddinge and were approved by Karolinska Institutet and the Linköpings ethics committee for animal research, Dnr 19604 − 2022. Animals were provided with food and water ad libitum, dark-light cycle, temperature and humidity were kept constant.

### Retrieval of mature and immature oocytes

Tissues collected from the mice were processed under a stereomicroscope (SMZ800N Nikon^®^). After dissection from the surrounding tissue, oviducts were transferred to M2 medium. The distended portion of the ampulla was punctured using Micro-Fine U-100 insulin syringes (0.3 mL, BD Medical) to release cumulus oocyte complex. Oocytes were denuded by repeated pipetting with a stripper and picked up. The ovaries were then mechanically isolated to release antral and large preantral follicles, and the oocytes were denuded by repeated pipetting with a stripper in M2 medium.

The collected oocytes (*n* = 47) were grouped based on their developmental stage under a stereomicroscope with following criteria: Mature (metaphase II, MII) oocytes with a visible polar body; Germinal vesicle (GV) oocytes with a visible germinal vesicle; Metaphase I (MI) oocytes showed neither a polar body nor a germinal vesicle. These oocytes were directly stained with MitoTracker to assess the distribution of mitochondria.

### Fluorescence and confocal microscopy

Oocytes were transferred into M2 medium containing 400 nM MitoTracker™ Red CMXRos (Invitrogen) for mitochondria labeling and incubated at 37 °C for 40 min. The oocytes were then transferred into fresh M2 medium and incubated at 37 °C for 15 min, twice. Subsequently, oocytes were fixed with 4% paraformaldehyde for 10 min and permeabilized with 0.5% Triton X-100 for 10 min. Finally, the oocytes were mounted on slides using antifade mounting media containing DAPI (Vectashield) and covered with coverslips. Prepared slides were stored at 4 °C until imaging.

Fluorescence images were captured using a confocal microscope (Zeiss) and analyzed with ImageJ (Fiji). Further computational analysis of the confocal images, including 3D surface rendering reconstructions and mitochondrial mass (surface rendering area) measurements, was performed using the Imaris software (Bitplane).

### Statistical analysis

The between-groups comparison was conducted using a two-tailed unpaired Student’s t-test. We considered differences between the groups to be statistically significant when the p-value was less than 0.05. The standard errors of the mean were calculated by web-based statistics software (http://www.endmemo.com/math/sd.php).

## Results

### Mitochondria exhibit a uniform distribution throughout the cytoplasm in mouse GV oocytes

**Fig. 1:**
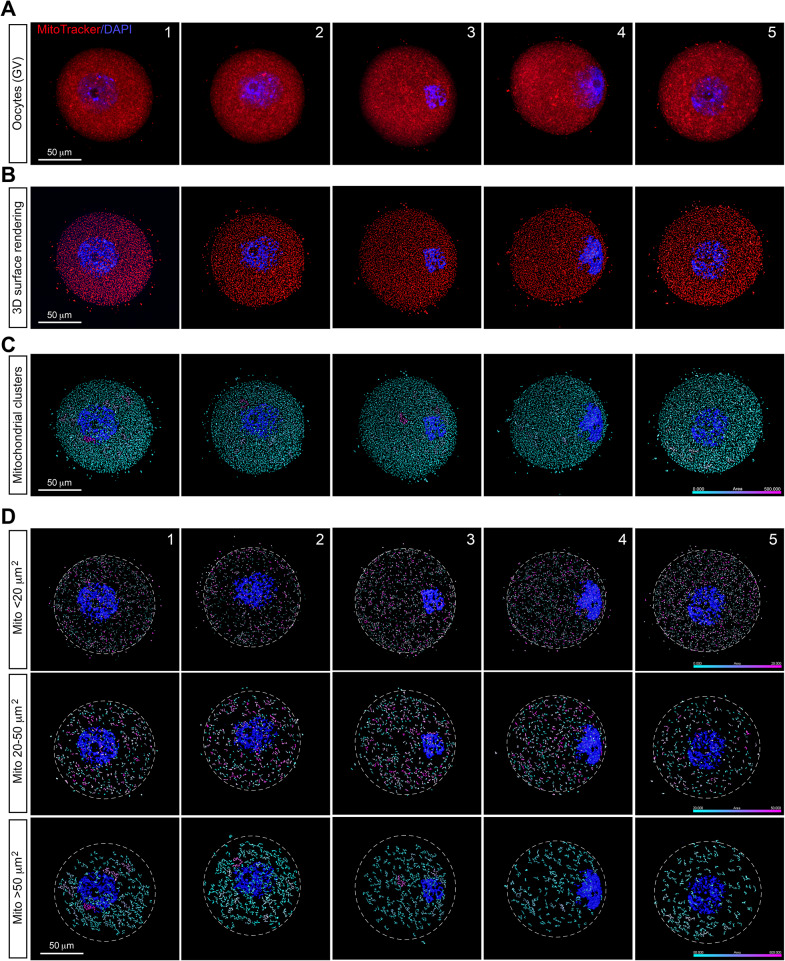
Mitochondrial distribution patterns in mouse GV oocytes. **A**. Representative confocal images depict mitochondria distribution patterns in five fresh mouse GV oocytes. Mitochondria in these oocytes were stained with MitoTracker (red), and nuclei were counterstained with DAPI (blue) to confirm meiotic stages. **B**. 3D surface renderings from the five GV oocytes in panel A show the spatial distribution atlas of mitochondria (red), with nuclei stained with DAPI (blue). **C**. Computational analysis of the distribution and size of individual mitochondria and mitochondrial clusters based on Panel B. The color scale indicates the size of individual mitochondria and mitochondrial clusters. **D**. The individual mitochondria and mitochondrial clusters in Panel C were categorized into three populations based on their surface rendering area: small population (rendering surface area < 20 µm^2^, the upper panel), medium population (rendering surface area between 20 and 50 µm^2^, the middle panel), and large population (rendering surface area > 50 µm^2^, the lower panel). The color scale indicates the size of disconnected individual mitochondria and mitochondrial clusters

Representative confocal images of the mitochondrial distribution profile in fresh mouse GV oocytes were presented in Fig. 1A. At the GV stage, spherical mitochondria were evenly distributed throughout the ooplasm. Further analysis using 3D surface rendering of confocal images confirmed this relatively even distribution of mitochondria in GV oocytes (Fig. 1B and C). Notably, alongside the uniform distribution, some mitochondria aggregated to form small clusters (Fig. 1C). The average size of the software-distinguishable individual mitochondria and mitochondrial clusters was approximately 19 µm^2^, with the largest mitochondrial clusters ranging from approximately 200 µm^2^ to 450 µm^2^ observed among different GV oocytes.

To systematically characterize the spatial distribution of mitochondrial clustering, we categorized mitochondria and their clusters into three groups basing on their sizes: a small population (rendering surface area < 20 µm^2^), a medium population (rendering surface area between 20 and 50 µm^2^) and a large population (rendering surface area > 50 µm^2^). This provides a deeper approach to investigate the distribution patterns of mitochondria and mitochondrial clusters within the specified size ranges. All three mitochondrial populations exhibited a relatively uniform distribution throughout the entire GV ooplasm (Fig. 1D). However, there was a tendency for the large population of mitochondrial clusters to be located more in the inner parts of ooplasm (Fig. 1D, lower panel). Very few huge mitochondrial clusters were observed in GV oocytes (pink clusters in the lower panel of Fig. 1D).

### Mitochondria aggregate to form clusters in MI mouse oocytes

**Fig. 2:**
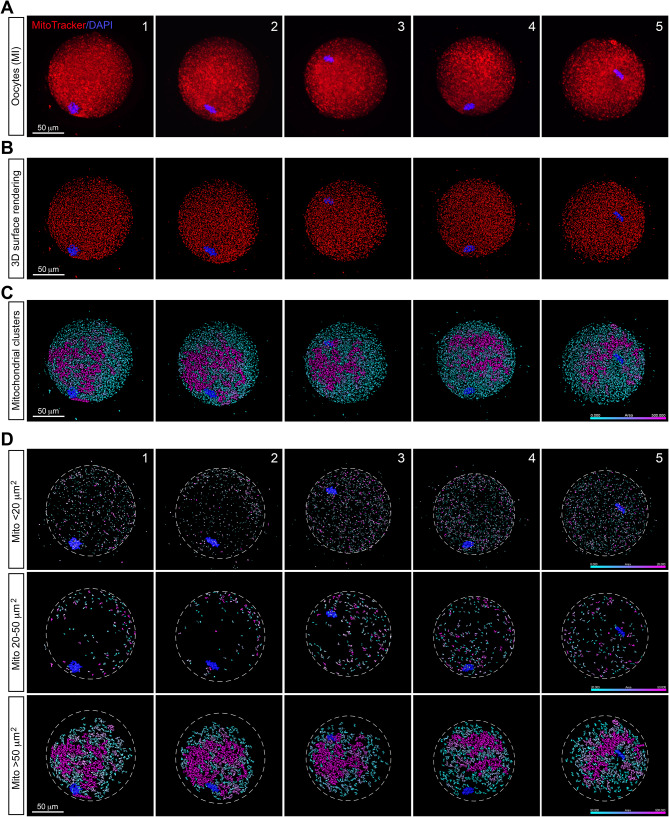
Mitochondrial distribution patterns in mouse MI oocytes. **A**. Representative confocal images show mitochondrial distribution patterns in five fresh mouse MI oocytes. Mitochondria were stained with MitoTracker (red), and chromosomes were counterstained with DAPI (blue) to confirm meiotic stages. **B**. 3D surface renderings from five MI oocytes in panel A show the spatial distribution atlas of mitochondria (red), with chromosomes stained with DAPI (blue). **C**. Computational analysis focuses on the distribution and size of individual mitochondria and mitochondrial clusters within MI oocytes based on panel B. The color scale indicates the size of disconnected individual mitochondria and mitochondrial clusters. **D**. The individual mitochondria and mitochondrial clusters in Panel C were categorized into three populations based on their surface rendering area: small population (rendering surface area < 20 µm^2^, upper panel), medium population (rendering surface area between 20 and 50 µm^2^, middle panel), and large population (rendering surface area > 50 µm^2^, lower panel). The color scale indicates the size of disconnected individual mitochondria and mitochondrial clusters

We next examined mitochondrial distribution in mouse oocytes at the MI stage. Representative confocal images of the mitochondrial distribution profile in fresh mouse MI oocytes along with their 3D surface renderings were shown in Fig. 2A and B, respectively. At this stage, mitochondria aggregated to form larger clusters, exhibiting distinct distribution patterns compared to those in GV oocytes. In MI oocytes, the average size of software-distinguishable individual mitochondria and mitochondrial clusters was approximately 30.8 µm^2^ (Fig. 2C). During the MI stage, the small mitochondrial population (rendering surface area < 20 µm^2^) maintained a relative homologous distribution across the entire ooplasm, albeit with minor variations among different oocytes (Fig. 2D, upper panel). The medium population (rendering surface area between 20 and 50 µm^2^) exhibited an uneven distribution within the cytoplasm of some oocytes, tended to aggregate to one side of the outer cytoplasm (Fig. 2D, middle panel). However, a distinct change was observed in the large population (rendering surface area > 50 µm^2^), which tended to aggregate to form larger clusters, with the largest clusters ranging from approximately 2000 µm^2^ to 5000 µm^2^ among different MI oocytes. These clusters were predominantly located toward the inner area of the cytoplasm (Fig. 2D, lower panel).

### Mitochondria progressively aggregate to form clusters in MII mouse oocytes

**Fig. 3:**
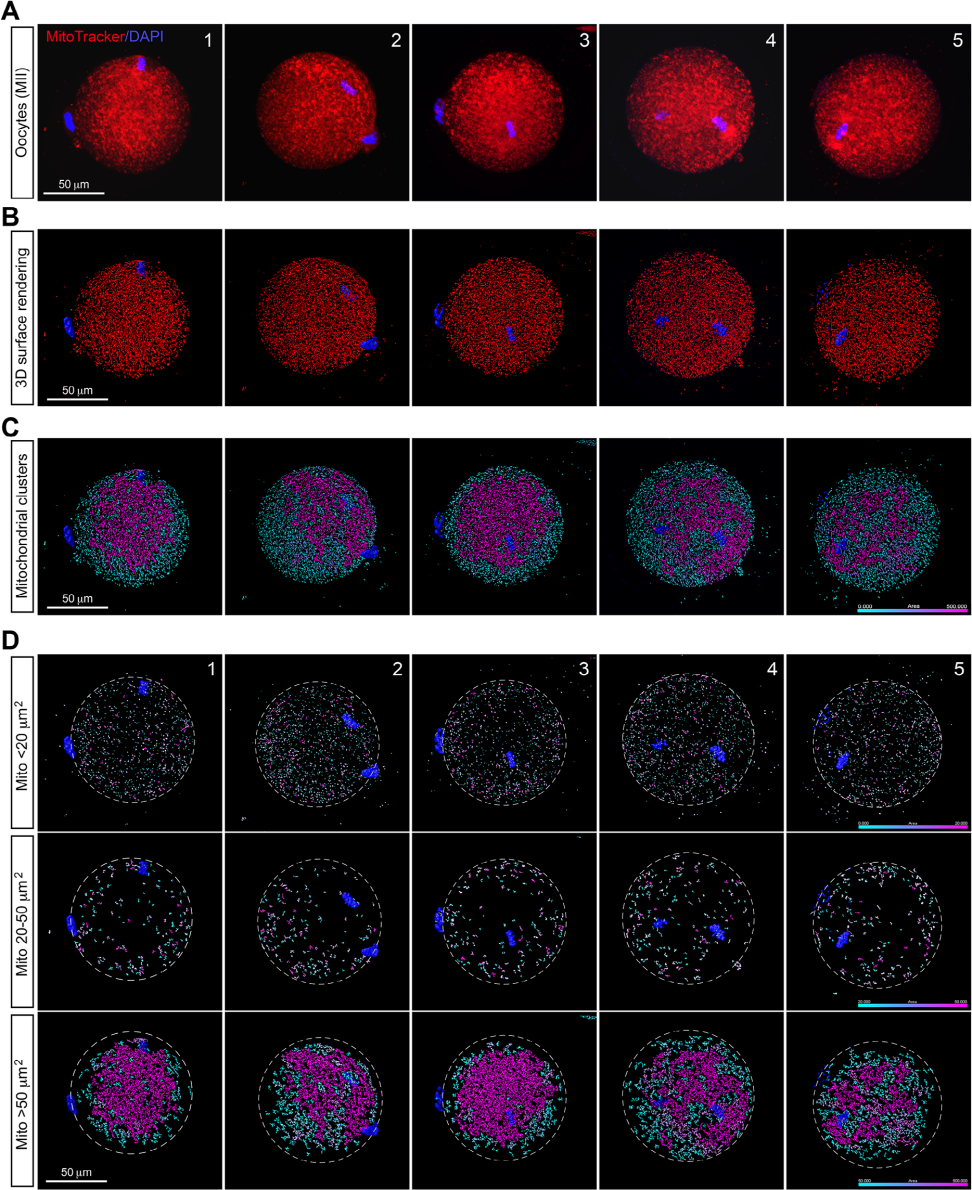
Mitochondrial distribution patterns in mouse MII oocytes. **A**. Representative confocal images show mitochondrial distribution patterns in five fresh mouse MII oocytes. Mitochondria are stained with MitoTracker (red), and chromosomes are counterstained with DAPI (blue) to confirm meiotic stages. **B**. 3D surface renderings from the five MII oocytes in panel A show the spatial distribution atlas of mitochondria (red), with chromosomes stained with DAPI (blue). **C**. Computational analysis of the distribution and size of individual mitochondria and mitochondrial clusters within MII oocytes based on panel B. The color scale indicates the size of disconnected individual mitochondria and mitochondrial clusters. **D**. The individual mitochondria and mitochondrial clusters in Panel C were categorized into three populations based on their surface rendering area: small population (rendering surface area < 20 µm^2^, upper panel), medium population (rendering surface area between 20 and 50 µm^2^, middle panel), and large population (rendering surface area > 50 µm^2^, lower panel). The color scale indicates the size of disconnected individual mitochondria and mitochondrial clusters

Representative confocal images of mitochondrial distribution and their 3D surface renderings of MII mouse oocytes stained with MitoTracker are presented in Fig. 3A and B. Comparing to MI oocytes, a similar distribution pattern of mitochondria in MII oocytes was observed (Fig. 3C and D). However, in MII oocytes, clustering of mitochondria became more pronounced and those clusters relocated toward the inner ooplasm (Fig. 3D, lower panel). Notably, the average size of software-distinguishable individual mitochondria and mitochondrial clusters increased to approximately 41.1 µm^2^. Correspondingly, there is a notable increase in the proportion of large mitochondrial clusters (> 50 µm^2^) in MII oocytes.

### Mitochondrial distribution in mouse oocytes undergoes significant reorganization throughout the GV, MI and MII stages

**Fig. 4:**
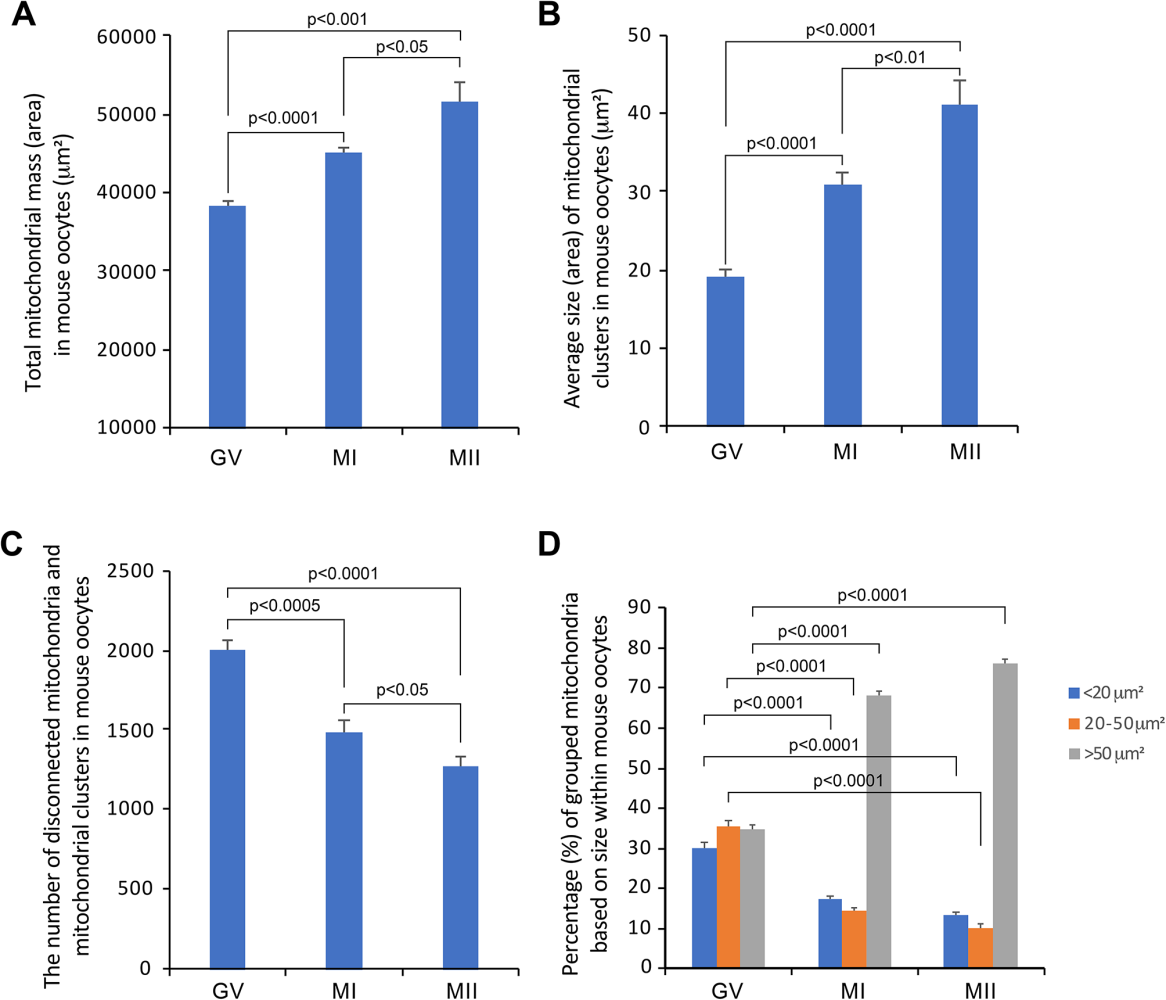
The distribution and organization of mitochondria within mouse oocytes undergo significant changes through different developmental stages. **A**. The average mitochondrial mass in mouse oocytes at the GV, MI and MII stages, quantified by the surface rendering area (µm^2^). **B**. The average size (µm^2^) of disconnected individual mitochondria and mitochondrial clusters in mouse oocytes across the GV, MI and MII stages. **C**. The average number of disconnected individual mitochondria and mitochondrial clusters in mouse oocytes at the GV to MI and MII stages. **D**. Percentages of small mitochondrial population (rendering surface area < 20 µm^2^), medium mitochondrial population (rendering surface area between 20 and 50 µm^2^), and large mitochondrial population (rendering surface area > 50 µm^2^) in relative to the total mitochondrial content in mouse GV, MI, MII oocytes. The numbers of oocytes analyzed in this figure were: GV (*n* = 6), MI (*n* = 8), MII (*n* = 7)

Our observations suggest that the distribution and behavior of mitochondria underwent significant changes with the oocytes progress from GV to MI and finally to MII. To delve deeper into these changes in a quantitative approach, mitochondrial content and average size were further analyzed in oocytes from different stages using the Imaris software. Mitochondrial content was estimated by analyzing the total rending surface area of mitochondria within each oocyte. As oocyte maturation proceeds, there was a substantial increase in mitochondrial mass from GV to MI and MII stages, as evidenced by the total area of mitochondria (Fig. 4A). Meanwhile, the average size of mitochondrial clusters also significantly increased during oocyte maturation from GV to MI and MII stages (Fig. 4B). Consequently, the total number of software-distinguishable disconnected individual mitochondria and mitochondrial clusters substantially decreased from GV to MI and MII stages (Fig. 4C).

To gain deeper insights into the dynamic changes of mitochondria during oocyte maturation, we meticulously quantified the proportion of small (rendering surface area < 20 µm^2^), medium (rendering surface area between 20 and 50 µm^2^) and large (rendering surface area > 50 µm^2^) mitochondrial cluster populations relative to the total mitochondrial content in oocytes at different stages. Our analysis revealed a substantial decrease in the small and medium mitochondrial cluster populations as oocyte maturation progressed. In contrast, the content of large mitochondrial cluster populations dramatically increased (Fig. 4D). These quantitative results were consistent with the observations in Figs. 1, 2 and 3.

## Discussion

During mammalian oocyte maturation, the mass of mitochondria increases, as evidenced by the total content of mitochondria shown in Fig. 4A of our study. This finding aligns well with previous reports indicating that mtDNA copy number significantly and progressively increases during the GV, MI and MII stages along the mouse oocyte maturation [39, 40]. Meanwhile, significant changes also occur in the distribution of mitochondria during this process [4]. The maintenance of proper mitochondrial redistribution is essential for oocyte quality [41]. Oocytes with abnormal mitochondrial dynamics and reduced cellular adenosine triphosphate (ATP) levels often exhibit poor developmental potential [10]. Despite our extensive knowledge, no comprehensive mitochondrial distribution atlas has been created for mouse oocytes during their maturation. Previous studies have utilized changes in mitochondria distribution, revealed by MitoTracker staining, as a biomarker for mitochondrial function to evaluate the quality of oocytes in control groups compared to those receiving specific interventions [28, 30, 32, 34, 35, 42]. Mitochondrial distribution has also been applied to explore the roles of mitochondrial dynamics-related proteins during mouse oocyte maturation [6, 29, 31, 33]. However, these studies have documented a wide range of mitochondrial distribution patterns in their respective normal control groups. This variability is evident not only across different studies but also among individual oocytes in the same control group. Furthermore, the criteria for defining homogeneous/even and clustered mitochondrial distribution patterns are vague and insufficient for reliably categorizing oocytes as ‘normal’ or ‘abnormal’. As a result, assessments that classify the mitochondrial distribution patterns in an oocyte as normal or abnormal are ambiguous and somewhat subjective, lacking a quantitative-based objective assessment for individual oocyte. This underscores the significance and necessity of creating a comprehensive atlas of the normal distribution pattern of mitochondria in mouse oocytes, serving as a biomarker for reflecting the oocyte condition and quality.

Our study revealed the distinct mitochondrial distribution patterns in mouse oocytes across different maturation stages with objective quantifications of the aggregation levels using a software. At the GV stage, individual mitochondria and small mitochondrial clusters are evenly distributed throughout the entire cytoplasm, lacking large mitochondrial clusters. At the MI stage, as oocytes transition from GV to MI, mitochondria aggregate into large clusters, primarily shifting toward the inner cytoplasm, but not specifically around the spindle. However, individual and small mitochondrial clusters remain uniformly distributed, albeit their numbers were reduced compared to the GV stage. At the MII stage, mitochondria continue to aggregate into even larger clusters in MII oocytes than in MI oocytes. Mitochondrial remodeling and clustering can influence mitochondrial function and distribution within the cell, impacting oocyte quality and developmental potential. Morpho-functional adaptations refer to changes in both the structure and function of mitochondria, which are essential for meeting the dynamic energy demands of the oocyte during its development. Understanding these adaptations is critical for improving reproductive outcomes, as they can affect the competency of oocyte in fertilization and further development to become a healthy embryo [10]. Therefore, we believe our findings are of fundamental importance in both basic and clinical research in female reproduction.

During literature review, we found that the distribution of mitochondria in mouse oocytes during maturation is a topic of debate. Some studies suggest that mitochondria in growing mouse oocytes are dispersed throughout the cytoplasm in small clusters but shift to a perinuclear region just before meiosis resumes. Following nuclear envelope breakdown, mitochondria form dense clusters around the MI spindle. In MII oocytes, mitochondria clusters redistribute into the cytoplasm (for reviews see [41]). Conversely, other studies indicate that from the GV stage until just before the germinal vesicle breakdown (GVBD), mitochondria preferentially accumulate near the perinuclear region. Post-GVBD and up to the MII stage, mitochondria are distributed evenly in the ooplasm (for reviews see [4, 9]). However, in our freshly collected mouse oocytes, we did not observe similar distributional alterations, neither the clustering to the perinuclear region in GV oocytes, nor the further clustering around the spindle in MI oocytes.

Emerging studies suggest that several in vitro culture conditions, interventions and treatments can significantly impact mitochondrial distribution in oocytes, such as oxygen concentration under in vitro culture conditions and hormones used during in vitro maturation (IVM) [43], culture media composition, temperature and pH [1], the process of vitrification or cryopreservation [44]. During literature review, we also found the variations in oocyte denudation methods across different studies. Some researchers employed 1 mg/mL of hyaluronidase [42], while others used different concentrations of hyaluronidase, such as 0.1% hyaluronidase [32], 0.3% hyaluronidase [30, 45], or 60 IU/ml hyaluronidase for 5 min, followed by the removal of zona pelucida [28]. Furthermore, some studies utilized mechanical methods such as repeated pipetting [6, 29], while others did not mention the denudation methods used [31, 34, 35]. Some studies also investigated the impact of different concentrations and exposure durations of hyaluronidase on oocytes, and found that high concentration and/or prolonged exposure time could compromise oocyte maturation, fertilization and developmental competence [46–50]. Notably, hyaluronidase has also been shown to negatively affect human cumulus cells during oocyte denudation [51]. Although it is still unclear how these diverse oocyte denudation methods employed in different studies impair mitochondrial distribution in oocytes at various stages, they might potentially impact oocyte quality, resulting in the inconsistent mitochondrial distribution patterns reported by different publications. Additionally, treatment of mouse oocytes with cisplatin leads to mitochondrial aggregation to the perinuclear region [34]. Collectively, all these studies imply that mitochondrial distribution in oocytes may be very sensitive to the environmental alterations, and even the change from in vivo to in vitro condition can cause their distributional alterations. Therefore, we performed this study using freshly collected oocytes, believing it is critical to build an atlas that closely resembles the in vivo situation and minimizes potential influencing factors.

In addition to mouse oocytes, previous studies have also investigated oocyte mitochondrial behaviors and distribution in other species, for instance, in bovine oocytes before and after in vitro maturation [37], in pig oocyte during in vitro maturation, fertilization and early embryo development [36], in zebrafish during oogenesis [38], and in human oocytes matured in vivo or in vitro and preimplantation embryos [25, 52]. Notably, a wide range of differences in mitochondrial aggregation and clustering patterns during oocyte maturation has been observed across these diverse species.

Taken together, our study established a mitochondrial atlas in mouse oocytes during maturation with an objective quantification of mitochondria in mouse oocytes at different maturation stage. Our findings suggest that beyond the increase in mtDNA copy number, mitochondria undergo dynamic re-organization during oocyte maturation. Mitochondrial clusters become progressively more pronounced and the clustered mitochondria move into the inner cytoplasm in mature oocytes. We propose that monitoring alterations in the spatiotemporal distribution of mitochondria within oocytes could provide a more refined approach to detect early responses of oocytes to external interventions. This could be particularly useful in observing the effects of specific treatments and optimizing ART-related protocols, such as culture medium choices, oocyte selection, supplementary factors tests, cryopreservation methods.

## Conclusions

Our study, for the first time, provided comprehensive mitochondrial distribution profiles in normal mouse oocytes at the GV, MI and MII stages using a combination of confocal images with 3D surface rendering, which were further quantitatively illustrated these mitochondrial distribution profiles via specialized Imaris software. Our findings reveal that mitochondria in mouse oocytes are highly dynamic, undergoing significant reorganization during oocyte maturation. The objective and standardized method we established is particularly useful for evaluating alterations in mitochondrial dynamics in oocytes. The mitochondrial distribution atlas we created can serve as a biomarker to monitor changes in oocyte condition and quality. While studies on oocyte mitochondrial dynamics have emerged in the field of female reproduction, particularly those involving ovarian follicles and oocytes, our approach offers a more precise quantitative method.

## Data Availability

No datasets were generated or analysed during the current study.

## Abbreviations

ART: Assisted Reproductive Technology
ATP: Adenosine Triphosphate
DOR: Diminished Ovarian Reserve
DRP1: Dynamin-Related Protein 1
FBS: Fetal Bovine Serum
FIS1: Mitochondrial Fission 1 Protein
GV: Germinal Vesicle
GVBD: Germinal Vesicle Breakdown
IVM: In Vitro Maturation
MFF: Mitochondrial Fission Factor
MFN1/2: Mitofusin 1/2
MI: Metaphase I
MIEF1/2: Mitochondrial Elongation Factor 1/2
MII: Metaphase II
mtDNA: Mitochondrial DNA
OPA1: Optic Atrophy Protein 1
OXPHOS: Oxidative Phosphorylation
ROS: Reactive Oxygen Species
3D: Three-Dimensional
